# Genomic and Proteomic Characterization of the Deltamethrin-Degrading Bacterium *Paracoccus* sp. P-2

**DOI:** 10.3390/microorganisms13112481

**Published:** 2025-10-30

**Authors:** Qing Li, Yawei Zhang, Xianfeng Ren, Qingguo Meng, Baocheng Xu, Lixia Fan, Changying Guo, Bingchun Zhang, Mingxiao Ning, Yutao Wang

**Affiliations:** 1 Laboratory of Quality and Safety Risk Assessment for Agro-Products of the Ministry of Agriculture (Jinan), Institute of Quality Standard and Testing Technology for Agro-Products, Shandong Academy of Agricultural Sciences, Jinan 250100, China; 232612009@njnu.edu.cn (Q.L.); m13792135609@163.com (Y.Z.); renxianfenga@163.com (X.R.); superdemeter@163.com (L.F.); cyguo808@163.com (C.G.); llzbest66@163.com (B.Z.); 2 Jiangsu Key Laboratory for Aquatic Crustacean Diseases, College of Marine Science and Engineering, Nanjing Normal University, 1 Wenyuan Road, Nanjing 210023, China; mlzzcld@aliyun.com; 3 College of Food and Bioengineering, Henan University of Science and Technology, Luoyang 471000, China; xbc76@163.com

**Keywords:** deltamethrin, *Paracoccus* sp., genomic, proteomic, biodegradation

## Abstract

Deltamethrin is widely employed for crop pest control, aquaculture pond clearance, and fish parasite treatment. Due to its photostability, thermal resistance, and lipophilicity, deltamethrin has a high potential for environmental persistence and bioaccumulation in aquatic organisms. This poses significant risks to aquatic ecosystems, the safety of aquatic products, and human health. Although our previous study isolated *Paracoccus* sp. P-2 from crab culture pond sediment and demonstrated its high efficiency in degrading deltamethrin, the underlying mechanisms and enzyme characteristics remain unelucidated. In this study, genomic analysis revealed that the *Paracoccus* sp. P-2 genome was assembled into 3 contigs with a total length of 4,451,812 bp, an average G + C content of 67.73%, and a total of 4462 predicted genes. In addition, a quantitative analysis of the *Paracoccus* sp. P-2 proteome identified 3052 proteins, with 2705 exhibiting significant differential abundance (FC ≥ 1.5 or FC ≤ 0.6667, and *p*-value ≤ 0.05) following deltamethrin exposure. Among them, many upregulated differentially expressed proteins were enriched in carbohydrate and energy metabolism pathways, indicating that *Paracoccus* sp. P-2 enhances its basal metabolic activity in response to deltamethrin-induced stress. More importantly, enzymes belonging to hydrolases, decarboxylases, and those involved in multiple xenobiotic metabolic pathways were upregulated and are likely to participate in the degradation of deltamethrin. This study elucidates the impact of deltamethrin on bacterial metabolism and its degradation mechanism by *Paracoccus* sp. P-2, providing crucial insights and microbial resources for researching pyrethroid biodegradation.

## 1. Introduction

Deltamethrin, which is classified as a Type II synthetic pyrethroid pesticide, has been widely applied in crop pest control, pond clearance in aquaculture, and treatment of various parasitic diseases in fish owing to its high efficacy and broad-spectrum activity [1,2]. However, with the increasing residual levels of deltamethrin in aquaculture waters, fishery pollution incidents have occurred frequently, leading to serious quality and safety issues in aquatic products [3]. Most pyrethroid pesticides are classified as environmental endocrine disruptors. They can be transmitted through the food chain and pose risks to human reproductive and physiological systems [4,5]. Some types may also have teratogenic, carcinogenic, and mutagenic effects [6,7,8]. To mitigate the environmental and public health risks associated with the use of pyrethroid pesticides, it is essential to develop rapid and effective methods for removing or reducing pesticide residues in the environment. Among various remediation approaches for contaminated environments, bioremediation based on the metabolic activity of pesticide-degrading microorganisms has emerged as one of the most promising and effective strategies for eliminating pesticide pollution. Microbial remediation offers advantages such as high efficiency, non-toxicity, environmental friendliness, ease of operation, and low cost [9,10].

Various microorganisms in nature, including bacteria, actinomycetes, and fungi, possess the ability to degrade deltamethrin [11]. In recent years, strains such as *Streptomyces rimosus* [12], *Lysinibacillus* sp. ZJ6 [13], and *Raoultella ornithinolytica-ZK4* [14] have been isolated from soil and shown to effectively reduce deltamethrin residues in vegetables and soil. In addition, our previous research led to the isolation of a deltamethrin-degrading bacterium (identified as *Paracoccus* sp. P-2) from a crab cultivation pond sludge that was frequently exposed to the deltamethrin [15]. This strain is the first *Paracoccus* species isolated from an aquatic environment that is capable of efficiently degrading deltamethrin. Nevertheless, the aquatic environment in which *Paracoccus* sp. P-2 thrives differs significantly from terrestrial settings. Compared with known terrestrial deltamethrin-degrading bacteria, it remains unknown whether this strain possesses similar high-efficiency degradation pathways and what the underlying mechanisms are.

During the degradation of pesticides, microorganisms secrete various specific enzymes that cleave the carboxylic ester bonds of pyrethroids, breaking them down into alcohols and small molecular carboxylic acids, and ultimately converting them into less toxic or non-toxic compounds [16,17]. To elucidate the specific enzymes involved in pesticide degradation by unknown degrading bacteria and their underlying mechanisms, genomics, proteomics and metabolomics based on sequencing technology and bioinformatics are widely employed as analytical tools. For instance, genomic analysis of *Pseudomonas putida* KT2440 revealed the involvement of multiple enzymes (e.g., oxygenases, oxidoreductases, cytochromes, dehydrogenases, efflux pumps, and glutathione S-transferases) in the metabolism of xenobiotics [18,19]. Kjeldal et al. [20] applied genomics, proteomics, and metabolomics to study the degradation mechanism of gemfibrozil by *Bacillus* sp. GeD10, identifying 284 proteins with more than twofold upregulation. Among these, cytochrome P450 hydroxylase, catechol dioxygenase, alcohol dehydrogenase, and glucuronidase were involved in drug metabolism. Guo et al. [21] reported that *Bacillus thuringiensis* could effectively remove deltamethrin within 48 h. Using proteomic and metabolomic approaches, their study revealed that during this process, the transcription of DNA and synthesis of heat shock proteins were inhibited, while decreased levels of oxalic acid and acetyl-CoA suppressed energy metabolism. Additionally, the strain resisted deltamethrin-induced oxidative stress by promoting endospore formation and germination. Although multi-omics approaches have been widely employed to elucidate the mechanisms of pesticide degradation in other microorganisms, they have not yet been applied to *Paracoccus* sp. P-2.

In this study, we first employed Nanopore sequencing (a third-generation technology) to decipher the complete genome of *Paracoccus* sp. P-2. Subsequently, based on a quantitative proteomic analysis, we investigated the response of *Paracoccus* sp. P-2 to deltamethrin stress by profiling differentially expressed proteins. Our study revealed significant alterations in cellular metabolism, particularly enhanced carbohydrate and energy metabolism, under pesticide-induced stress. Furthermore, we identified key enzymatic features involved in deltamethrin degradation, including upregulation of hydrolases, decarboxylases, and enzymes related to xenobiotic metabolic pathways. The results advance the characterization of *Paracoccus* sp. P-2, support further studies into pyrethroid biodegradation, and aid in the assessment of environmental safety.

## 2. Materials and Methods

### 2.1. Bacterial Strain and Chemicals

The bacterial strain *Paracoccus* sp. P-2 was previously isolated in our laboratory [15]. Grade deltamethrin (25 g/L) was purchased from Zhongbao Green Agriculture Group, Plant Protection Institute, Chinese Academy of Agricultural Sciences (Beijing, China). The components of Luria–Bertani medium (LB) included 10.0 g/L peptone, 5.0 g/L yeast extract and 10.0 g/L NaCl. The basic salt medium (MCM) contained: 1.0 g/L NH_4_NO_3_, 0.5 g/L MgSO_4_·7H_2_O, 0.5 g/L (NH_4_)_2_SO_4_, 0.5 g/L KH_2_PO_4_, 0.5 g/L NaCl, 1.5 g/L K_2_HPO_4_ and 0.05 g/L yeast extract. All media were adjusted to pH 7.0 and autoclaved at 121 °C for 20 min.

### 2.2. Genomic DNA Extraction and Sequencing

Extracting genomic DNA using the SDS method. Approximately 0.3 g of cell pellet was pulverized in liquid nitrogen and transferred to a 15 mL tube containing 8 mL of SDS lysis buffer (BioFroxx 3250GR500, Einhausen, Germany). The mixture was incubated at 50 °C for 1.5 h with gentle inversion every 20 min. After incubation, the lysate was cooled to room temperature and centrifuged at 10,000× *g* for 10 min. The supernatant was collected and sequentially extracted with equal volumes of phenol: chloroform: isoamyl alcohol (25:24:1) and then chloroform: isoamyl alcohol (24:1). Following each extraction, the sample was centrifuged at 10,000× *g* for 10 min. The resulting aqueous supernatant was transferred, and DNA was precipitated by adding 0.8 volumes of isopropanol. The DNA pellet was washed twice with ice-cold 75% ethanol, air-dried, and subsequently dissolved in 100 μL of EB buffer (Qiagen 19086, Venlo, The Netherlands). To remove RNA, the sample was treated with 3 μL of RNase (100 mg/mL) at 37 °C for 30 min. The DNA was further purified using 0.5× volume of magnetic beads and its quality was assessed using Nanodrop, Qubit, and agarose gel electrophoresis. After quality assessment and purification, a 1D library was constructed using the SQK-LSK110 kit (Oxford Nanopore Technologies, Oxford, UK) according to the manufacturer’s instructions. A short-insert library was prepared using the VAHTS^®^ Universal Plus DNA Library Prep Kit for MGI V2/for Illumina V2 (Vazyme, Nanjing, China). Qualified libraries were sequenced on the Nanopore PromethION and Illumina NovaSeq 6000 platforms.

### 2.3. Genome Annotation and Analysis

The raw sequencing data first underwent quality control to filter out low-quality and excessively short reads. De novo genome assembly was then performed, followed by error correction of the initial draft genome. Based on the polished assembly, structural annotation was carried out to identify genomic features including repetitive sequences, coding genes, non-coding RNAs, genomic islands, and CRISPR elements. For functional annotation, a systematic analysis was conducted using eight general databases (Pfam, Refseq, Uniprot, Nr, Tigrfams, COG, KEGG, GO) and several specialized databases (ARDB, CAZy, CARD, CYP450, VFDB, TCDB), along with signal peptide prediction.

### 2.4. Culture Conditions and Sample Preparation

*Paracoccus* sp. P-2 was activated and cultured by LB medium, and the bacteria liquid in logarithmic growth phase was collected by shaking culture at 37 °C and 200 r/min for 12 h. The bacteria liquid was centrifuged at −4 °C, 10,000 r/min for 1 min, the supernatant was discarded, and 1 mL MCM medium was added to re-suspend the bacteria. The cleaning process was repeated twice to completely remove the residual LB medium. Finally, 6 mL of bacteria liquid re-suspended in MCM was obtained. 1 mL of the above *Paracoccus* sp. P-2 solution was inoculated into a test tube containing 5 mL of MCM medium, and the final concentration of deltamethrin solution was added to 200 mg/L. The inoculation solution was placed in a shaker at 30 °C and 200 r/min for 48 h. MCM medium without deltamethrin under the same culture conditions was used as blank control. All experiments in three copies. After the cultivation, the bacterial solution was centrifuged (10,000 r/min, 1 min, −4 °C), and the supernatant and bacterial precipitate were collected for subsequent deltamethrin concentration determination and omics analysis.

### 2.5. Detect the Content of Deltamethrin by Gas Chromatography

Based on the method described by Ning et al. [15], 1 mL of culture solution was extracted with 2 mL of acetone and 2 mL of n-hexane by ultrasonication for 20 min, followed by centrifugation at 6000 rpm for 5 min. The upper organic phase was collected, and the extraction was repeated twice. The combined extracts were concentrated under a stream of N_2_ at 70 °C, reconstituted to 1 mL with n-hexane, and purified on a Florisil column pre-conditioned with n-hexane and acetone (9:1, *v*/*v*). The eluate was collected, evaporated to dryness under N_2_, and diluted 100-fold with n-hexane. Analysis was carried out using a GC system equipped with a DB-1 non-polar capillary column (30 m × 0.25 mm × 0.25 μm) and an electron capture detector (ECD). The injector and detector temperatures were set at 240 °C and 300 °C, respectively, with an injection volume of 1 μL and a constant carrier gas (N_2_) flow rate of 1.2 mL/min. The oven temperature program was: 80 °C (1 min), raised to 230 °C at 15 °C/min (hold 1 min), then to 270 °C at 40 °C/min (hold 10 min). Nitrogen was used as the carrier gas. Sensitivity was evaluated through the limit of detection (LOD). Based on the signal-to-noise ratio, the LOD and LOQ for deltamethrin in MCM medium samples were determined to be 0.01 mg/kg. Final sample quantification adopted external standard calibration.

### 2.6. Proteomic Sample Preparation and LC-MS/MS Analysis

#### 2.6.1. Protein Extraction

The cell pellet was thawed on ice and resuspended in an appropriate volume of lysis buffer (8 M urea, 1 mM PMSF, 2 mM EDTA). The suspension was sonicated on ice for 5 min and then centrifuged at 15,000× *g* for 10 min at 4 °C. The supernatant was collected, and the protein concentration was determined using a BCA assay kit (Beyotime Biotechnology, Shanghai, China).

#### 2.6.2. Protein Digestion and Desalting

A 100 μg aliquot of protein was diluted to 200 μL with 8 M urea, reduced with 5 mM DTT at 37 °C for 45 min, and alkylated with 11 mM iodoacetamide in the dark at room temperature for 15 min. Then, 800 μL of 25 mM ammonium bicarbonate and 2 μL of trypsin (Promega, V5280, Madison, WI, USA) were added, and the mixture was digested overnight at 37 °C. The resulting peptides were acidified to pH 2–3 with 20% TFA and desalted using a C18 column (Millipore, Billerica, MA, USA). Peptide concentration was quantified using the Pierce^TM^ Quantitative Peptide Assay Kit (Thermo Fisher, Waltham, MA, USA).

#### 2.6.3. LC-MS/MS Analysis

Peptides were separated on a Vanquish Neo UHPLC nanoflow system (Thermo Fisher, Waltham, MA, USA). Mobile phase A was 0.1% formic acid in water, and mobile phase B was 0.1% formic acid in acetonitrile (100%). A trap-analytical dual-column setup was used: PepMap Neo Trap Cartridge (300 μm × 5 mm, 5 μm) and an Easy-Spray^TM^ PepMap^TM^ Neo UHPLC analytical column (150 μm × 15 cm, 2 μm) (Thermo Fisher Scientific, Waltham, MA, USA). The analytical column was maintained at 55 °C. A total of 200 ng of peptides was loaded at a flow rate of 2.5 μL/min with an effective gradient of 6.9 min and a total run time of 8 min.

Data-independent acquisition (DIA) was performed on an Orbitrap Astral mass spectrometer (Thermo Scientific, Waltham, MA, USA) in positive ion mode. Full MS scans covered 380–980 *m*/*z* at a resolution of 240,000 (at 200 *m*/*z*), with a normalized AGC target of 500% and maximum injection time of 5 ms. MS^2^ spectra were acquired in DIA mode with 299 scanning windows, an isolation window of 2 Th, HCD collision energy of 25%, normalized AGC target of 500%, and maximum injection time of 3 ms.

### 2.7. Bioinformatics Analysis

DIA data were processed using DIA-NN (v1.8.1) in library-free mode with the following parameters: UniProtKB database taxonomy ID 265 (*Paracoccus* genus, 22 May 2025 release, 277,282 entries); deep learning-based spectral library prediction enabled; match-between-runs (MBR) enabled to generate spectral libraries from DIA data for requantification. Both precursor and protein-level false discovery rates (FDR) were filtered at 1%. The filtered data were subsequently subjected to bioinformatic analysis. Identified proteins and differentially expressed proteins (DEPs) were systematically annotated and enriched through Gene Ontology (GO) terms and KEGG pathways.

## 3. Results and Discussion

### 3.1. Genome Sequencing of Paracoccus *sp.* P-2

**Figure 1:**
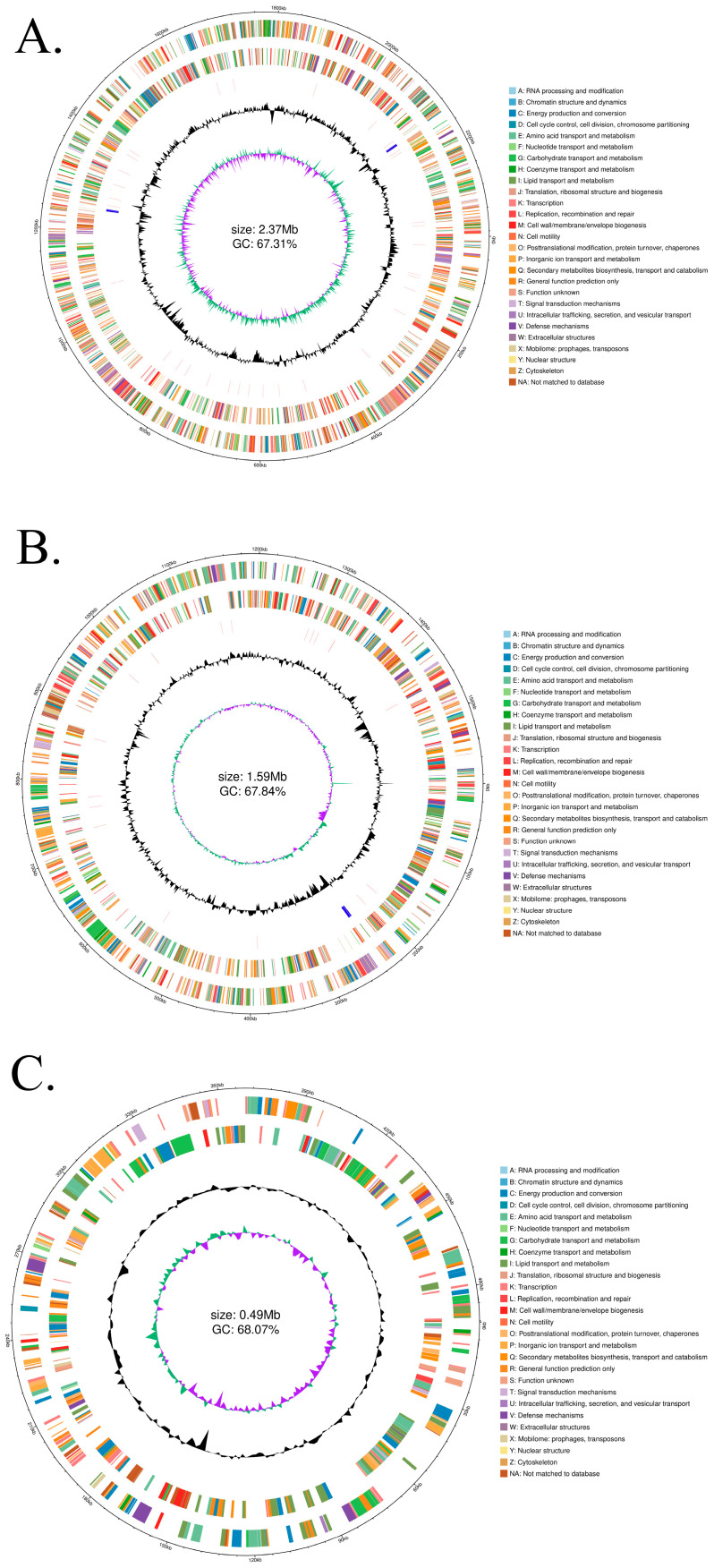
Circular map of the complete genome of *Paracoccus* sp. P-2. The genome comprises two confirmed chromosomes (**A**,**B**) and a third small circular molecule (**C**), whose biological nature-whether chromosomal or plasmid—has not yet been classified. Notes (from outer to inner circle): The first circle (outermost): Genomic coordinates. The second circle: Genes on the positive strand, colored according to their COG functional categories. The third circle: Genes on the negative strand, similarly colored by COG. The fourth circle: Locations of rRNA (blue) and tRNA (red) genes. The fifth circle: GC content (calculated with a 2000 bp sliding window). The sixth circle (innermost): GC skew (calculated with a 2000 bp sliding window), with green and purple indicating positive and negative values, respectively.

The genome assembly yielded three circular DNA molecules with a total size of 4,451,812 bp and average G  +  C content of 67.73%. A total of 4462 genes were predicted, including 59 tRNA operons and 444 pseudogenes, with other gene categories detailed in Table S1. The circular view of the genome from the R package circlize (0.4.12) [22] is presented in Figure 1. The circular view showed the genomic coordinates, the positive and negative strand of the gene, the rRNA and tRNA on the genomic sequence and the G  +  C content of the genome (Figure 1). The genome sequences of *Paracoccus* sp. P-2 are accessible in SRP with accession numbers PRJNA1336399. SRA records will be accessible with the following link after the indicated release date: (accessible date after 1 January 2026).

### 3.2. Genome Functional Annotation

**Figure 2:**
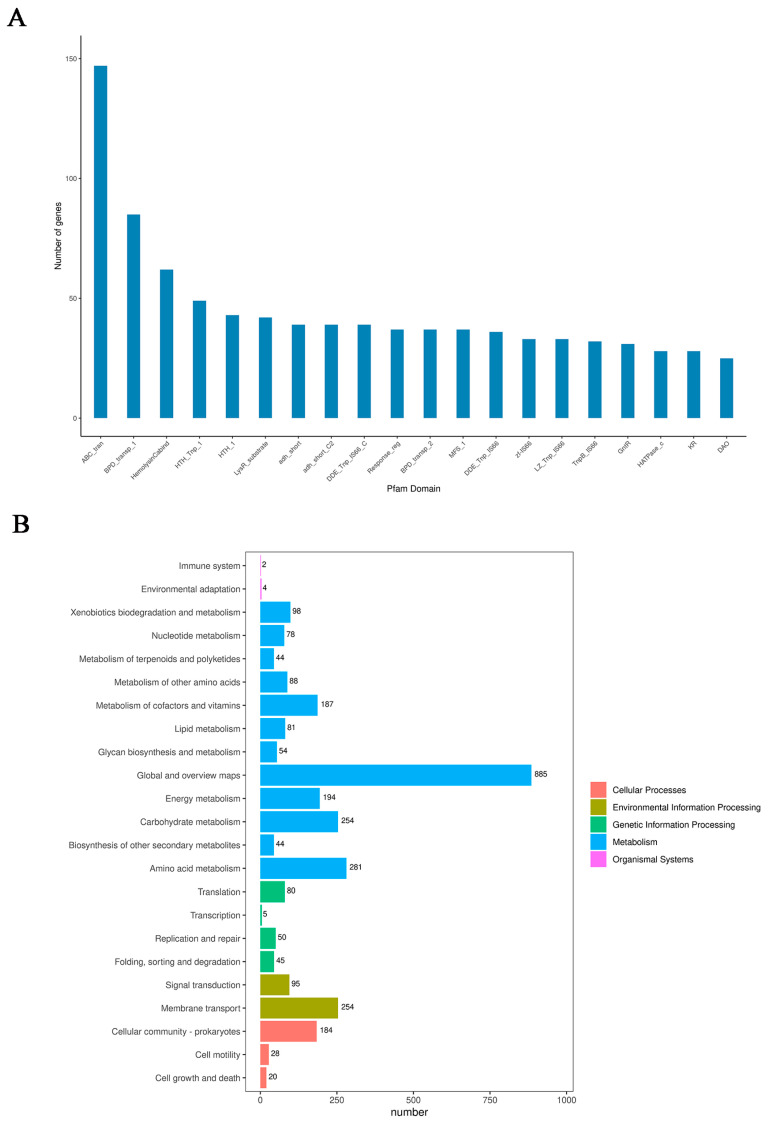
Functional annotation of the genome. (**A**) Distribution of identified protein domains based on the PFAM database. (**B**) Categorization of enriched KEGG pathways.

To obtain comprehensive gene function information, we performed gene function annotation using eight major databases, including UniProt [23], KEGG [24] and KEGG Pathway, GO [25], Pfam [26], COG [27], TIGERfams [28], RefSeq, and NR. The predicted gene sequences were aligned against functional databases such as COG, KEGG, UniProt, and RefSeq using BLAST+ (Version: 2.11.0+), resulting in the gene function annotation outcomes. The statistical results of the annotations are shown in Table S2. Among the top 20 domains annotated based on Pfam, we observed that genes belonging to “ABC transporters” were the most abundant (Figure 2A). As we know, ABC transporters comprise a large superfamily of proteins that facilitate the translocation of a diverse array of substrates, including ions and macromolecules, across cellular membranes through ATP binding and hydrolysis. Furthermore, these transporters are also critically involved in the cellular uptake and efflux of numerous organic pollutants [29,30]. These results suggest that the abundance of ABC transporter genes provides *Paracoccus* sp. P-2 with a significant capacity for deltamethrin transport.

As shown in Figure 2B, the genes annotated by KEGG are divided into 5 major categories and 23 minor subcategories. Among them, the subcategory with the largest number of genes is metabolic pathways, which include amino acid metabolism, carbohydrate metabolism, energy metabolism, metabolism of cofactors and vitamins, among others. The abundance of genes related to metabolic pathways provides the fundamental basis for the survival and deltamethrin degradation capability of *Paracoccus* sp. P-2. It is worth noting that a substantial number (254) of membrane transporter genes have been annotated in the genome of *Paracoccus* sp. P-2. Pollutants are often adsorbed onto the microbial membrane surface and subsequently internalized into the cells—a process in which membrane transporter proteins play a crucial role [31]. This abundance of transporter genes highlights the strain’s strong capacity for pollutant transport.

### 3.3. Changes in Deltamethrin on the Protein Expression of Paracoccus *sp.* P-2

**Figure 3:**
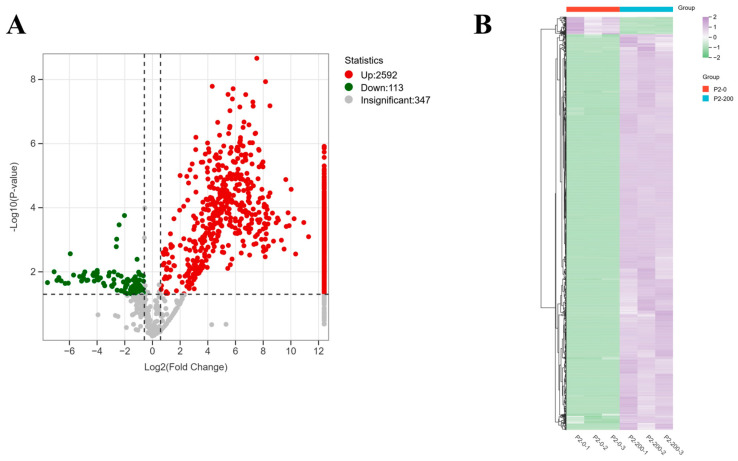
Differentially expressed proteins (DEPs) in the control and treatment samples. (**A**) Volcano picture of DEPs (red represents up-regulated proteins, green represents down-regulated proteins, and gray represents unaltered proteins). (**B**) Hierarchical clustering of DEPs.

The results in Figure S1 show that *Paracoccus* sp. P-2 reduced the concentration of deltamethrin in the culture medium by 60.55% within 48 h. Proteins were extracted from three biologically replicated *Paracoccus* sp. P-2 cultures, cultured without or with deltamethrin, and analyzed by DIA quantitative proteomics (Figure 3A). The mass spectrometry proteomics data have been deposited to the ProteomeXchange Consortium (https://proteomecentral.proteomexchange.org, accessed on 9 October 2025) via the iProX partner repository with the dataset identifier PXD068977. A total of 3052 proteins were identified, of which 2705 proteins showed significant abundance differences (FC ≥ 1.5 or FC ≤ 0.6667, and *p*-value ≤ 0.05) (Table S3). Of these differentially expressed proteins (DEPs), deltamethrin exposure up-regulated 2592 and down-regulated 113 compared to the control. In order to further demonstrate the expression pattern of differential proteins, hierarchical clustering analysis was performed on all DEPs. The results showed that the expression profile of DEPs could clearly distinguish between the treatment group and the control group, indicating that deltamethrin had a significant effect on *Paracoccus* sp. P-2 (Figure 3B).

**Figure 4:**
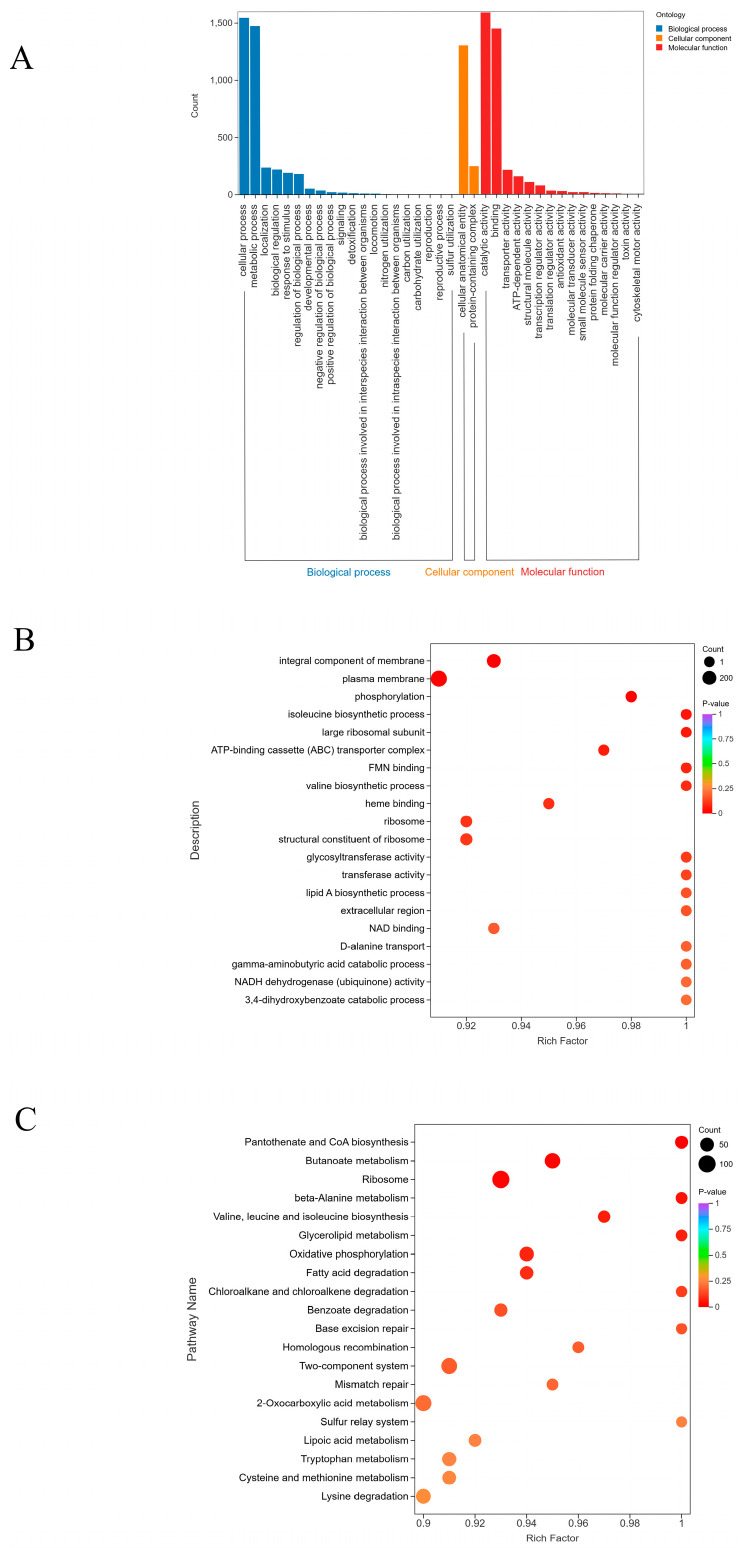
Gene Ontology (GO) categories and Kyoto Encyclopedia of Genes and Genomes (KEGG) categories for DEPs. (**A**) GO classification analysis of DEPs in level two. (**B**) GO enrichment analysis of DEPs. (**C**) KEGG enrichment analysis of DEPs.

In order to analyze the function of DEPs, we performed GO annotation and enrichment analysis. The results (Figure 4A,B) showed that DEPs were annotated into three categories: biological process (BP), cellular component (CC) and molecular function (MF), which were classified into 37 functional groups. In the biological process category, most DEPs focus on cellular process and metabolic process. In the cell component category, cellular anatomical entity is the main annotation item; in the molecular function category, binding and catalytic activity are the main ones. In addition, DEPs were significantly enriched in multiple GO terms, including integral components of the membrane, plasma membrane, phosphorylation, and isoleucine biosynthetic process. Subsequently, KEGG pathway enrichment analysis of 2705 DEPs showed that these proteins were enriched in 120 pathways, mainly involved in pantothenate and CoA biosynthesis, butanoate metabolism, ribosome and beta-alanine metabolism (Figure 4C).

### 3.4. Effect of Deltamethrin on Carbon Metabolism in Paracoccus *sp.* P-2

In MCM medium, trace yeast extract was only used to support the initial survival of *Paracoccus* sp. P-2, and its growth and metabolism mainly depended on deltamethrin as carbon source. Therefore, it is particularly important to analyze the metabolic response mechanism of *Paracoccus* sp. P-2 under deltamethrin stress. Carbon metabolism, as the core pathway of energy generation and biosynthesis precursor supply, mainly includes glycolysis and gluconeogenesis, pentose phosphate pathway (PPP) and Entner-Doudoroff (ED) pathway [32]. Among them, glycolysis and PPP are ubiquitous in all areas of life. Glycolysis is mainly responsible for the degradation of glucose to pyruvate, resulting in ATP and a variety of biosynthetic precursors [33,34]. Under deltamethrin stress, *Paracoccus* sp. P-2 exhibited upregulated expression of 56 proteins in the Glycolysis/Gluconeogenesis pathway, including key enzymes such as 2,3-bisphosphoglycerate-independent phosphoglycerate mutase, Glucokinase, Glucose-6-phosphate isomerase, and fructose-bisphosphate aldolase (Table S4). PPP, as a metabolic pathway parallel to glycolysis, provides NADPH and ribose-5-phosphate as two key metabolites for cell biosynthesis and antioxidant defense system [35,36,37]. Glucose-6-phosphate 1-dehydrogenase and 6-phosphogluconolactonase in this pathway also showed up-regulation.

The tricarboxylic acid cycle (TCA cycle), as the final step in the complete oxidation of glucose under aerobic conditions, oxidizes acetyl-CoA (derived from the metabolism of amino acids, fatty acids, and carbohydrates) to CO_2_ and H_2_O, and stores the released energy in reduced coenzymes (NADH, FADH_2_), eventually generating ATP [38,39]. The cycle starts from the condensation of acetyl-CoA and oxaloacetic acid catalyzed by citrate synthase to form citrate, which is then isomerized to isocitrate by aconitase. Isocitric acid was decarboxylated and oxidized to α-ketoglutaric acid under the action of isocitrate dehydrogenase. The latter was then subjected to oxidative decarboxylation catalyzed by the α-ketoglutarate dehydrogenase complex (composed of α-ketoglutarate dehydrogenase (E1), dihydrolipoic acid transsuccinate (E2) and dihydrolipoic acid dehydrogenase (E3)) to generate succinyl coenzyme A; then succinyl-CoA is converted into succinic acid by succinyl-CoA synthetase, and succinic acid is oxidized by succinate dehydrogenase to produce fumaric acid; fumaric acid is hydrated by fumaric acid enzyme to generate malic acid. Finally, malic acid is regenerated into oxaloacetate under the action of malate dehydrogenase to complete the cycle [40]. Proteomic analysis showed that deltamethrin stress induced the up-regulation of 48 proteins in TCA cycle in *Paracoccus* sp. P-2. They include Isocitrate dehydrogenase NADP, Pyruvate dehydrogenase E1 component alpha subunit, Acetyltransferase component of pyruvate dehydrogenase complex, Dihydrolipoyl dehydrogenase, Succinate dehydrogenase iron–sulfur subunit, fumarate hydratase class II and Malate dehydrogenase (Table S4). These results suggest that deltamethrin exposure stimulates carbon metabolism in *Paracoccus* sp. P-2.

### 3.5. Effect of Deltamethrin on Energy Metabolism in Paracoccus *sp.* P-2

Methane metabolism, sulfur metabolism and nitrogen metabolism, as the key bridges connecting inorganic environment and organic organisms, are the biochemical basis for organisms to maintain life activities and construct their own structures [41]. This study found that certain proteins showed significant change in their expression levels during energy metabolism. Key enzymes involved in methane metabolism (total 47 up-regulated proteins, e.g., 2,3-bisphosphoglycerate-independent phosphoglycerate mutase), sulfur metabolism (total 41 up-regulated proteins, e.g., thiosulfate/3-mercaptopyruvate sulfurtransferase), and carbon fixation via the Calvin cycle (total 31 up-regulated proteins, e.g., pyruvate, phosphate dikinase) all exhibited significantly up-regulated expression. In the process of nitrogen assimilation in nitrogen metabolism, glutamine synthesis pathway and glutamate dehydrogenase pathway are the two main ways to absorb inorganic nitrogen (such as NO_3_^−^ and NH_4_^+^) and convert it into organic nitrogen-containing substances [42]. In this study, the expression levels of glutamine synthetase, L-glutamine synthetase, glutamate dehydrogenase and glutamate synthase large subunit involved in these two pathways were significantly increased (Table S4).

In the process of electron transfer, energy is used to pump protons out of the cell membrane to form a proton gradient, thereby promoting ATP synthesis. This process begins with the formation of NAD (P), H^+^ and electrons from NADH under the action of NADH dehydrogenase, and the formation of fumaric acid and electrons from succinic acid (FADH_2_) under the catalysis of succinic dehydrogenase. Subsequently, electrons are transferred to cytochrome C reductase by coenzyme Q (CoQ), and finally to cytochrome c oxidase by cytochrome C [43]. This study found that a series of proteins related to this process, including NADH dehydrogenase subunit E, NAD (P)/FAD-dependent oxidoreductase, Succinate dehydrogenase iron–sulfur subunit, C-type cytochrome and Cytochrome B, were significantly up-regulated (Table S4). We speculate that *Paracoccus* sp. P-2 enhances its energy storage capacity by up-regulating the expression of electron transport chain-related proteins to maintain cell life activities under deltamethrin stress. In organisms, the main source of ATP is oxidative phosphorylation. As a coupling reaction, oxidative phosphorylation can release energy to promote the synthesis of ATP by ADP and inorganic phosphate through the respiratory chain [44]. It has been found in tumor cell studies that it enhances energy supply by overexpressing ATP synthase [45]. This study identified 55 up-regulated proteins involved in the oxidative phosphorylation pathway, such as ADP/GDP-polyphosphate phosphotransferase and ATP synthase epsilon chain, were also significantly up-regulated (Table S4), which further proved that the ATP synthesis ability of *Paracoccus* sp. P-2 strain was enhanced under deltamethrin stress. The above results showed that deltamethrin stress could induce the enhancement of the energy metabolism of *Paracoccus* sp. P-2.

### 3.6. Biodegradation of Deltamethrin by Paracoccus *sp.* P-2

Under deltamethrin stress, *Paracoccus* sp. P-2 exhibited not only alterations in carbon and energy metabolism but also significant upregulation of multiple key proteins associated with deltamethrin biodegradation, as identified among the differentially expressed proteins and further supported by annotation analysis of KEGG pathways and structural domains. Microbial biodegradation of pollutants often begins with cellular chemotaxis, whereby the pollutant is adsorbed onto the microbial cell surface [46]. Studies have shown that hydrophobic proteins such as pili, fibrils, and outer membrane proteins (OMPs) can enhance cell surface hydrophobicity (CSH), thereby promoting the adsorption of hydrophobic organic pollutants like deltamethrin [47,48]. The marked upregulation of flagellin (Table S5) suggests that *Paracoccus* sp. P-2 may enhance its motility to actively migrate toward deltamethrin-contaminated sites, thereby establishing the spatial foundation for subsequent degradation reactions. After locating the pollutant, the primary challenge becomes the internalization of the hydrophobic deltamethrin macromolecules. Transmembrane transport is a key step in microbial degradation, indicating that the passage of hydrophobic substrates through the cell membrane is also a crucial stage in the biodegradation process [49,50]. The upregulation of most members of the ABC transporter family (total 135 proteins) and transmembrane proteins (Table S5) offers a solution, as they are likely responsible for actively recognizing and transporting deltamethrin molecules across the membrane, overcoming the energy barrier of substrate uptake and enabling efficient intracellular accumulation of the degradation substrate [29,51].

Upon entering the cell, the core degradation process relies on the catalytic activity of enzymatic systems. The upregulation of hydrolases—such as carboxylesterase, guanine deaminase, cell wall hydrolase, and amidohydrolase (Table S2)—likely facilitates the cleavage of the ester bond in deltamethrin, leading to initial detoxification and the formation of primary metabolites including 3-phenoxybenzoic acid (PBA). This step serves as the rate-limiting stage for complete degradation and is critical for the elimination of the pesticide’s toxicity [43,52]. To facilitate the thorough mineralization of metabolic intermediates, the pronounced upregulation of decarboxylase expression likely catalyzes the decarboxylation of aromatic intermediates (e.g., PBA), yielding simpler metabolites such as benzoate that are more susceptible to further catabolism. These compounds are subsequently assimilated into central carbon metabolic pathways, achieving complete pollutant decomposition [53]. Furthermore, elevated expression levels were detected for key enzymes involved in multiple xenobiotic metabolic pathways (total 71 up-regulated proteins), including the degradation of benzoate, chloroalkane/chloroalkene, naphthalene, and polycyclic aromatic hydrocarbons (Table S5). This proteomic profile suggests that *Paracoccus* sp. P-2 likely mobilizes aromatic compound metabolic networks during deltamethrin degradation, facilitating efficient removal of pesticide residues and their metabolic intermediates through coordinated multi-enzyme activity [43]. These results indicate that *Paracoccus* sp. P-2 demonstrates significant potential for application in the bioremediation of deltamethrin-contaminated environments.

## 4. Conclusions

This study provides a multi-omics analysis of *Paracoccus* sp. P-2, revealing its efficient deltamethrin degradation mechanism. Genomic analysis revealed key genes encoding proteins essential for cellular metabolism and substrate transport, the presence of which provides the genetic foundation for the degradation of deltamethrin by *Paracoccus* sp. P-2. Proteomic data further indicated that under deltamethrin stress, *Paracoccus* sp. P-2 upregulates proteins associated with carbohydrate and energy metabolism, thereby enhancing its metabolic activity to cope with the stress and providing essential support for the degradation of deltamethrin. Moreover, the induction of key enzymes, including hydrolases, decarboxylases, and xenobiotic-metabolizing enzymes, suggests that *Paracoccus* sp. P-2 mobilizes aromatic compound metabolic networks during deltamethrin degradation, thereby facilitating the efficient removal of both pesticide residues and their metabolic intermediates through coordinated multi-enzyme activity. These findings offer valuable insights into microbial bioremediation and highlight the potential of *Paracoccus* sp. P-2 for application in mitigating environmental pyrethroid contamination.

## Data Availability

The original contributions presented in this study are included in the article/Supplementary Materials. Further inquiries can be directed to the corresponding authors.

## Supplementary Materials

The following supporting information can be downloaded at: https://www.mdpi.com/article/10.3390/microorganisms13112481/s1, Figure S1: Degradation of deltamethrin by *Paracoccus* sp. P-2; Table S1: Statistical results of gene prediction; Table S2: Statistical results of gene annotation encoding; Table S3: Overview of differentially abundant proteins; Table S4: A list of some of the important DEPs which involved in energy metabolism; Table S5: A list of some of the important DEPs which involved in biodegradation of deltamethrin by *Paraccocus* sp. P-2.
