# Supplementary material for: Genomic and Proteomic Characterization of the Deltamethrin-Degrading Bacterium Paracoccus sp. P-2
Source: Microorganisms. 2025 Oct 30;13(11):2481. doi: 10.3390/microorganisms13112481 (PMC12654547; doi:10.3390/microorganisms13112481)
Supplement: Supplementary file 1 [file microorganisms-13-02481-s001.zip › Table S1.pdf]

Table S1. Statistical Results of Gene Prediction

Type	Number	Total_len	Average_len	Percentage of genome(%)
Gene	4,462	4,011,611	899	90.11
CDS	4,362	3,989,343	915	89.61
tRNA	59	4,713	80	0.11
23S rRNA	3	8,475	2,825	0.19
16S rRNA	3	4,380	1,460	0.10
5S rRNA	3	327	109	0.01
tmRNA	1	331	331	0.01
misc_rna	31	4,042	130	0.09
