# Supplementary material for: Genomic and Proteomic Characterization of the Deltamethrin-Degrading Bacterium Paracoccus sp. P-2
Source: Microorganisms. 2025 Oct 30;13(11):2481. doi: 10.3390/microorganisms13112481 (PMC12654547; doi:10.3390/microorganisms13112481)
Supplement: Supplementary file 1 [file microorganisms-13-02481-s001.zip › Table S2.pdf]

Table S2. Statistical Results of Gene Annotation Encoding

Item	Count	Percentage
All	4,362	100.00%
Annotation	4,354	99.82%
KEGG	2,528	57.96%
Pathway	1,503	34.46%
Nr	4,352	99.77%
Uniprot	4,332	99.31%
GO	3,271	74.99%
COG	3,810	87.35%
Pfam	3,711	85.08%
Refseq	4,321	99.06%
Tigerfam	2,434	55.80%
