# Supplementary figures and images for: Genomic and Proteomic Characterization of the Deltamethrin-Degrading Bacterium Paracoccus sp. P-2
Source: Microorganisms. 2025 Oct 30;13(11):2481. doi: 10.3390/microorganisms13112481 (PMC12654547; doi:10.3390/microorganisms13112481)

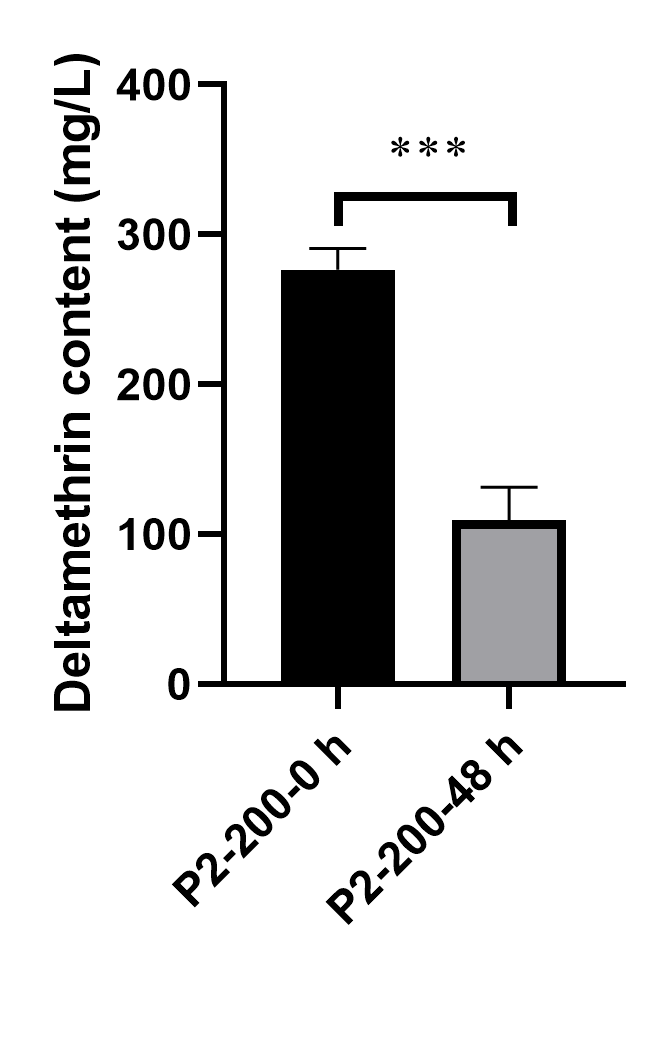

Supplement: Supplementary file 1 [file microorganisms-13-02481-s001.zip › Figure S1.png]
